# Vascular co‐option and vasculogenic mimicry mediate resistance to antiangiogenic strategies

**DOI:** 10.1002/cnr2.1318

**Published:** 2020-12-09

**Authors:** Francesco Pezzella, Domenico Ribatti

**Affiliations:** ^1^ Nuffield Division of Laboratory Science, Radcliffe Department of Medicine John Radcliffe Hospital, University of Oxford Oxford UK; ^2^ Department of Basic Medical Sciences, Neurosciences and Sensory Organs University of Bari Medical School Bari Italy

**Keywords:** angiogenesis, antiangiogenesis, tumor growth, vascular co‐option, vasculogenic mimicry

## Abstract

**Background:**

The concept that all the tumors need the formation of new vessels to grow inspired the hypothesis that inhibition of angiogenesis would have led to “cure” cancer. The expectancy that this type of therapy would have avoided the insurgence of resistance was based on the concept that targeting normal vessels, instead of the cancer cells which easily develop new mutations, would have allowed evasion of drug caused selection is, however, more complex as it was made apparent by the discovery of nonangiogenic tumors. At the same time an increasing number of trials with antiangiogenic drugs were coming out as not as successful as expected, mostly because of the appearance of unexpected resistance.

**Recent Findings:**

Among the several different mechanisms of resistance to antiangiogenic treatment by now described, we review the evidences that vascular co‐option and vasculogenic mimicry by nonangiogenic tumors are effectively two of such mechanisms. We focused on reviewing exclusively the study, both clinical and preclinical, that offer a demonstration that vascular co‐option and vasculogenic mimicry are effectively two mechanisms of both intrinsic and acquired resistance.

**Conclusion:**

The discovery that vascular co‐opting and vasculogenic mimicry are two ways of escaping antiangiogenic treatment, prompts the need for a better understanding of this phenomenon in order to improve cancer treatment.

## 1 INTRODUCTION

In the last decades, a large body of work has led to propose that the formation of new vessels, also known as angiogenesis, is necessary for a neoplasm to growth beyond the diameter of a few millimeters. Following a first period in which data from all over the world seemed to support this hypothesis, “inducing angiogenesis” has been considered to be a hallmark of cancer.^1^

The idea that angiogenesis is necessary to cancer growth is the brainchild of Judah Folkman who went as far as to propose that, by inhibiting angiogenesis with appropriate drugs, it would be possible to treat any type of tumors or, at least, maintaining or inducing dormancy and preventing the formation of metastatic lesions.^2^ The production of drugs which can actually block effectively angiogenesis in vitro and in preclinical animal model has led to a rush of large clinical trials which, in the beginning, had wide resonance.^3^ As data started to come through it was, however, quite evident that this was not going to be the cases as antiangiogenic treatment turned out to provide, at the best, some very modest positive effects.^4^, ^5^, ^6^, ^7^ Contrary to expectation it became clear that resistance to antiangiogenic treatment did occur, the question was: which are the mechanisms?

**FIGURE 1:**
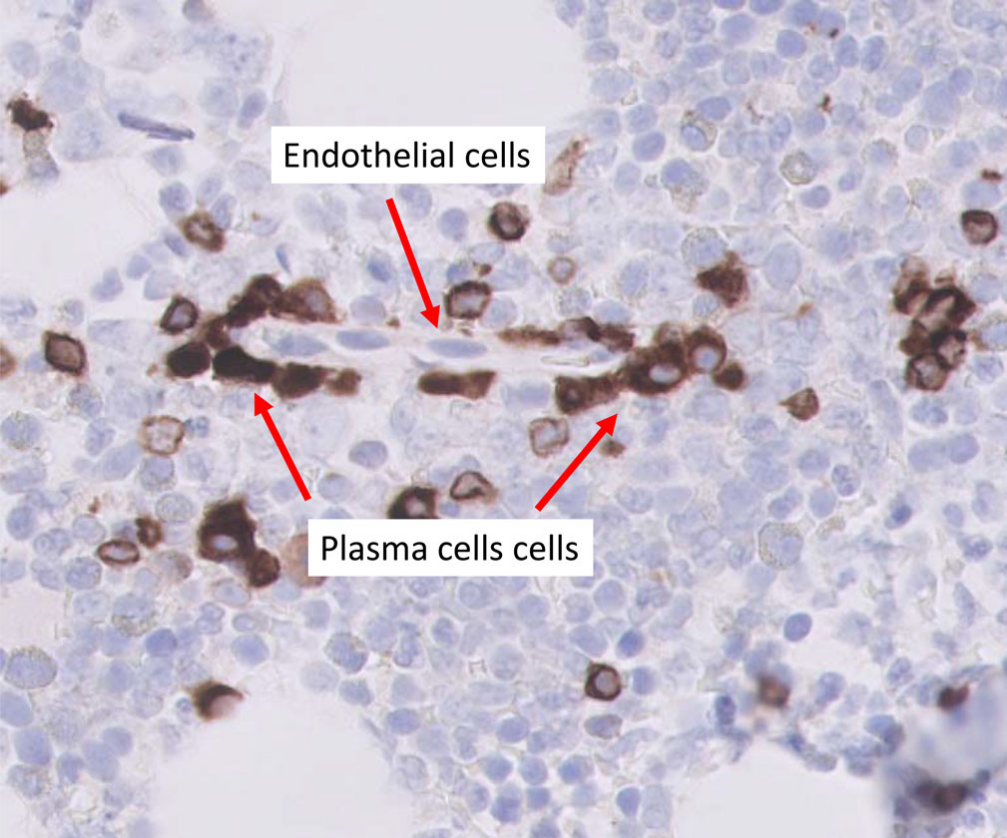
An example of physiological vascular co‐option: normal bone marrow. Immunostain with anti‐CD79a antibody (dark brown). Nuclear counterstain with hematoxylin. Marrow blood vessels are co‐opted by a subpopulation of plasma cells organize in a single cell line

**FIGURE 2:**
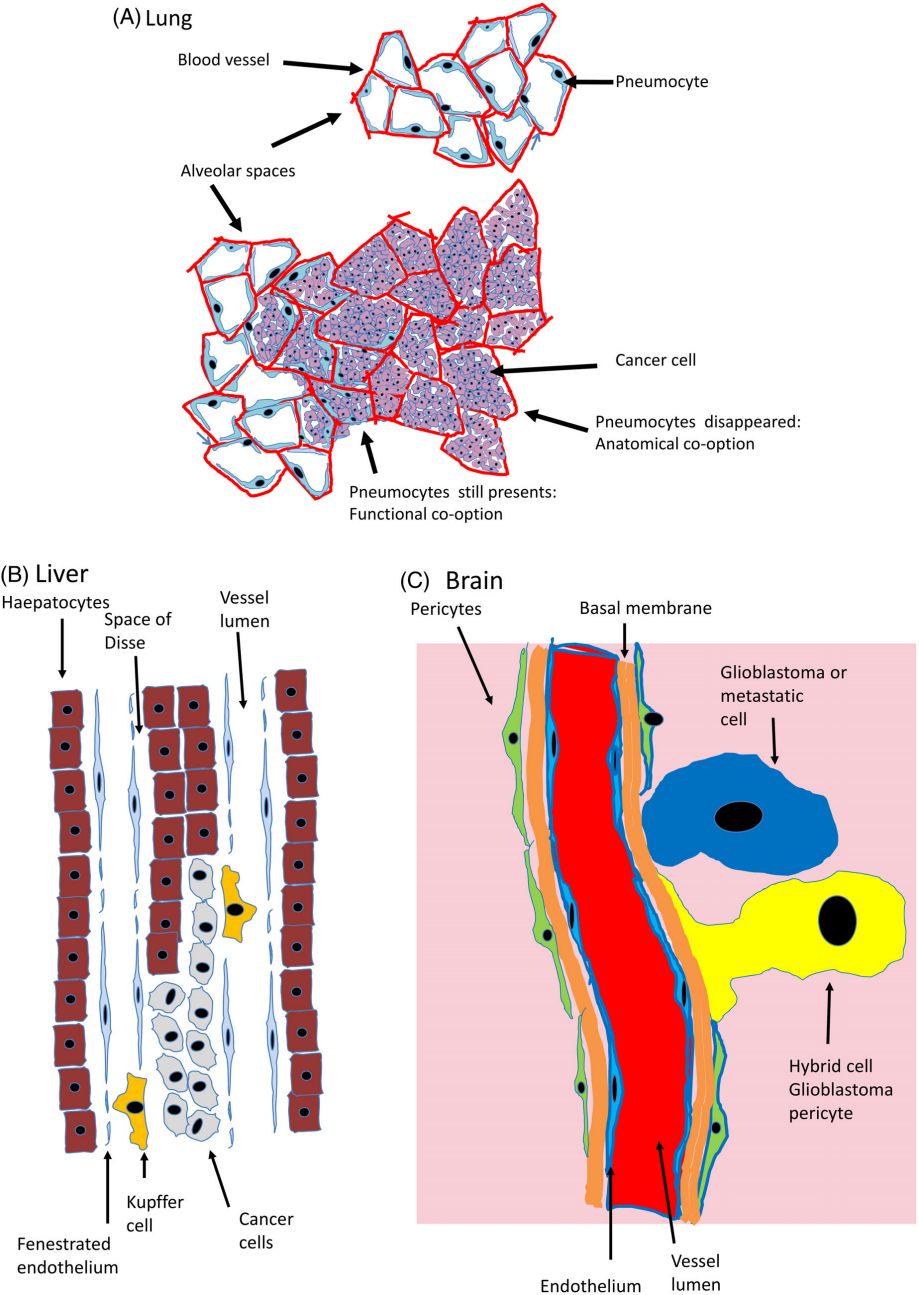
Different co‐option modalities. A, Mechanisms of co‐option: the lung. Anatomical and functional co‐option. In the normal lung: the alveolar spaces are lined by the pneumocytes which separate the air space from the vessels. In nonangiogenic cancer growth the tumor cells are filling the alveoli. Initially, the pneumocytes are still in place so the co‐option is functional as the cancer cells are not in direct contact with the vessels. On the right, a deeper portion of the tumor: the pneumocytes have been detached from the abluminal surface of the vessels and have disappeared. The cancer cells now are in direct contact with the vessels and the co‐option is not only functional but also anatomical. B, Liver: Liver sinusoid are vascular spaces in which a space (space of Disse) is present between endothelium and underlying liver cells. In this organ, the neoplastic cells exploit the liver sinusoids not by anatomically linking to the vascular structure but by taking the place of the hepatocytes (replacement). C, In the brain neoplastic both glioblastoma or metastatic cells can co‐opt the vessels directly (blue neoplastic cells). The glioblastoma cells can also co‐opt by merging and forming hybrid cells with the pericytes (yellow hybrid cell)

Resistance to antiangiogenic treatments had been first predicted in 1997^8^ when an unexpected mechanism of cancer growth had been discovered: growth of nonangiogenic tumors which exploited the preexisting normal vessels^9^ by co‐opting them,^10^ rather than induce, and rely upon, newly formed one.^8^ Angiogenesis is defined as “the process of new blood‐vessel growth”^11^ and an angiogenic tumor is one in which the cancer cells induces angiogenesis in order to grow.^12^ A nonangiogenic tumor is defined as one not inducing the formation of new nonneoplastic blood vessels.^9^ Vascular co‐option was firstly defined as a mechanism by which nonangiogenic cancer cells obtain a blood supply by hijacking the existing vasculature.^13^, ^14^ However, as shown in an example (Figure 1), co‐option is a physiological phenomenon as, for example, sub population of plasma cells co‐opt vessels in normal bone marrow.^15^ We are, therefore, proposing a more comprehensive definition of vascular co‐option, including normal and neoplastic cells, which is “the establishment of a stable anatomical and/or functional interaction between the co‐opting cell and the co‐opted blood vessels” (Figure 2).

Vascular mimicry also occurs in tumors not inducing the formation of new nonneoplastic blood vessels, that is, nonangiogenic. However, these nonangiogenic cancer cells instead of co‐opting preexisting vessels, form themselves channels that provide blood flow to the neoplastic mass.^16^

This patterns of tumor vascularization are commonly ignored but can mediate cancer progression and establishment of metastasis.

## 2 EVIDENCES FROM CLINICAL EXPERIENCE

As said above, for many years the data seemed to support Folkman's hypothesis. A first group of studies supporting it reported an association between the microvascular density (MVD) present in tumors and their aggressiveness,^17^ an hypothesis successively challenged.^18^ The discovery of the vascular endothelial growth factor (VEGF) family of proteins and their receptors,^19^, ^20^ their crucial involvement in inducing angiogenesis both in neoplastic and nonneoplastic conditions, plus their presence in a variety of tumors^21^, ^22^, ^23^, ^24^ provided more support. In 1996, the identification of angiostatin and its antiangiogenic efficacy in murine models, further seemed to confirm the fact that this approach could treat patients with both early and advanced malignancies.^25^, ^26^ At the time the idea developed that, because the targeted vessels were not neoplastic by themselves, as they were normal new vessels growing inside the neoplastic mass, this type of cancer treatment could be immune from resistance.^27^

Contrary to expectations the clinical trials results have been disheartening. Representative is what has been observed in high‐grade glioma: overall survival failed to improve with antiangiogenic drugs.^28^ However, in these patients, bevacizumab treatment can relieve symptoms by reducing the severity of intracranial oedema.^29^ In advanced breast cancer, improvement in disease‐free but crucially not overall survival has been seen while the results in early breast tumors are inconclusive.^30^ Antiangiogenic treatment has instead improved the results in patients with metastatic colorectal cancer,^31^ still the benefits achieved are in the range of months rather than years. No benefit has been instead achieved for patients with early colorectal cancer.^32^ Disappointing the results in small cell lung cancer^33^ while positive results have been reported in non‐small cell lung cancer, although the gained progression free and overall survival is once again limited.^34^

## 3 MECHANISMS OF RESISTANCE

It becomes evident that resistance to antiangiogenic treatment is widespread. It can be intrinsic, when it is observed at the beginning of the treatment, or acquired, that is, that it affects the relapsing disease after an initial response to therapy.^35^, ^36^ Following the first description of a putative mechanism, that is, the nonangiogenic cancer growth and progression by exploiting the preexisting vessels,^8^ a number of different mechanisms of resistance started to be discovered and has been reviewed elsewhere.^37^ This article is focused on the nonangiogenic tumor growth as a resistance mechanism.

## 4 NONANGIOGENIC TUMORS AND VASCULAR CO‐OPTION

Nonangiogenic tumors grow in the absence of angiogenesis by two main mechanisms. One way is by cancer cells infiltrating and occupying the normal tissues to exploit preexisting vessels, which is known as vascular co‐option or vessel co‐option. The second is one, less frequent, in which no new vessels are formed but the cancer cells themselves forms channels able to provide blood flow, the so called vasculogenic mimicry.^9^

Nonangiogenic tumors can be very aggressive and induce metastatic spreading but as they lack new vessels, it was immediately evident that antiangiogenic treatment could not have worked in these patients as the target of the treatment itself is not present.^8^

In 1999, Holash et al reported that tumor cells migrate to host organ blood vessels in sites of metastases, or in organs with an extensive vascularization likes the brain, and initiate nonangiogenic tumor growth instead of classic angiogenesis. These vessels then regress owing to apoptosis of the constituent endothelial cell, apparently mediated by angiopoietin‐ 2 (Ang‐2). Lastly, at the periphery of the growing tumor mass angiogenesis occurs by cooperative interaction of VEGF and Ang‐2. Tumor cells often have immediate access to blood vessels, such as when they metastasize to or are implanted within a vascularized tissue, co‐opt and often grow as cuffs around adjacent existing vessels.^13^ A host defense mechanism is activated, in which the co‐opted vessels trigger an apoptotic cascade, probably by autocrine induction of Ang‐2, followed by vessel regression resulting in tumor death. However, successful tumors overcome this vessel regression by initiating neoangiogenesis.^38^

The ability of cancer cells to co‐opt the vessels initiates the process through few of them will intravasate, starting the metastatic process.^39^

Maniotis et al^16^ illustrated a novel way of formation of vascular channels by human melanoma cells and named it “vasculogenic mimicry” to highlight de novo generation of vascular channels without the participation of endothelial cell and independent of angiogenesis. Transcriptomics of aggressive vs more indolent human cutaneous melanoma cell lines demonstrated an increase in the expression of laminin 5 and matrix metalloproteinases‐1, ‐2, and ‐9 (MMP‐1, MMP‐2, MMP‐9) and MT1‐MMP in the highly aggressive cells,^40^ suggesting that their interaction with the extracellular microenvironment is different from that of the more indolent cells, and that increased expression of MMP‐2 and MT1‐MMP along with matrix deposition of laminin 5 are required for their mimicry. Vasculogenic mimicry can serve as a marker for tumor metastasis, a poor prognosis, worse survival, and a highest risk of cancer recurrence.

## 5 CLINICAL STUDIES

Only a limited number of retrospective clinical studies looking at the correlation between the presence of nonangiogenic tumors and the effect of antiangiogenic drugs has been so far published (Table 1).

**TABLE 1:** Clinical studies demonstrating that vascular co‐option is associated with resistance to antiangiogenic treatment summary

Paper	Tumor investigated	Treatment received	Results	Type of resistance
Frentzas et al^41^	Liver, metastatic colorectal cancer	Neoadjuvant bevacizumab	Failure to respond associated with nonangiogenic pattern	Failure to respond—Intrinsic
Lazaris et al^42^	Colorectal cancer liver metastases	Neoadjuvant chemiotherapy plus bevacizumab	Major reduction of vessels in angiogenic compared to nonangiogenic lesions	Failure to respond—Intrinsic
Verhoeff et al^29^	Primary glioblastoma	Bevacizumab	Nonangiogenic growth at post mortem	Failure to respond—Intrinsic
di Tomaso et al^43^	Primary glioblastoma	Cediniranib	Nonangiogenic growth in relapse post cediniranib	Relapse post first response—Acquired
de Groot et al^44^	Primary glioblastoma recurrent	Bevacizumab	Nonangiogenic growth in patients failing to respond	Failure to respond—Intrinsic
Falchetti et al^45^	Primary glioblastoma recurrent	Bevacizumab	Nonangiogenic growth in relapse post bevacizumab	Relapse post first response—Acquired
Mehta et al^46^	Primary breast cancer	Bevacizumab	Lack of response with no radiological evidences of vascular changes	Failure to respond—Intrinsic
Helfrich et al^47^	Melanoma metastases	Bevacizumab	Growth during treatment	Failure to respond—Intrinsic
Kats‐Urgurlu et al^48^	Renal cell carcinoma metastatic to the lung	Cediranib	Nonangiogenic growth	Relapse post first response—Acquired
Yagi et al^49^	Lung carcinoma	Surgical	Possible spreading through the lung of nonangiogenic tumors	Hypothetical primary resistance to surgical treatment

Frentzas et al^41^ studied two groups of patients. The first one with primary colorectal cancer which developed liver metastases. These were treated with neoadjuvant bevacizumab and then underwent complete resection of the metastases. The second group was made up by breast cancer patients with liver metastases and was composed by two cohorts: one treated with neoadjuvant chemotherapy and bevacizumab and the second with chemotherapy alone. Patients were scored by the pathologist after resection as good or poor responder according to the ratio between viable tissue and necrosis. In patients with metastatic colorectal cancer an higher number of nonangiogenic lesions was found in the viable tissue of the poor responder while prevalently angiogenic metastases were seen among the good responders. At follow up, the patients with angiogenic residual viable tissues had a longer overall survival than the one with prevalently nonangiogenic secondaries. The authors than investigated two cohorts of breast cancer patients with liver metastases, a setting in which results with antiangiogenic therapy have been disappointing: one treated with bevacizumab chemotherapy and one with chemotherapy alone prior to metastases resection: all but one, out of the 17 metastatic lesions examined, were nonangiogenic.^41^

Lazaris et al^42^ investigated colorectal cancer liver metastases, from three cohorts: one not receiving any neoadjuvant, a second receiving neoadjuvant chemotherapy and a third treated with chemotherapy plus bevacizumab. Upon treatment with chemotherapy and bevacizumab, the nonangiogenic lesions showed no or minimal necrosis (range of viable cells 90%‐100%) while the angiogenic tumors had no more than 2% of viable cells (on which the scoring was done). A similar pattern was seen in the patients treated with chemotherapy alone, the only differences being that in the angiogenic tumors the percentage of viable tissue was higher (between 5% and 30%). Untreated nonangiogenic metastases were comparable to the treated one, while untreated angiogenic had a more variable amount of necrosis. This study support the hypothesis that nonangiogenic tumors are not only more resistant to bevacizumab but also to the neoadjuvant chemotherapy used (the drugs used are not specified by the authors).^42^

The lymph node is also involved in nonangiogenic growth by vascular co‐option.^50^ Jeong et al^51^ confirmed this finding and described also how in node metastases from colorectal cancer patients treated or not treated with bevacizumab, no differences in the intratumor vascularization where found.

Antiangiogenic treatment of primary central nervous systems tumors has been used for several years but, again, the results have not lived up the expectations.^29^ Post mortem histological examination of patients died after receiving treatment with cediranib, an inhibitor of VEGF receptor 2 (VEGFR2) tyrosine kinases,^43^ or bevacizumab regimen^44^ showed that the glioma cells were growing around preexisting vessels in a nonangiogenic fashion. In two case reports, vascular co‐option and progression in absence of angiogenesis in human brain tumor samples, surgical and autoptic, has been illustrated.^29^, ^45^

Breast cancer patients treated with Bevacizumab showed, by dynamic contrast‐enhanced magnetic resonance (DCE‐MRI) imaging, three response patterns. In the first one, a decrease in K‐trans values over the extent of the tumor was demonstrated, that is, decreased vascular permeability and/or vascular surface area, while the second was characterized by extensive necrosis. These two patterns indicate a response to antiangiogenic treatment. In the third one, instead, no changes were seen and the authors conclude that, in these lesions, the vessels were independent of VEGF. Whether they were preexisting normal vessels or not, it has not been investigated in this study^46^ and therefore we can only say that nonangiogenic growth may be, but not necessarily, responsible for the resistance to treatment.

Helfrich et al investigated the vascularization of metastases from stage III melanoma patients enrolled in the United Kingdom adjuvant bevacizumab trial. All the metastatic lesions presenting in subjects treated with bevacizumab, had a mature vessel morphology and phenotype in contrast with the newly formed vessels present in the relapsing disease from the trial patients not receiving the antiangiogenic drug. The authors do not discuss whether these are preexisting vessels or by now matured “normalized” newly formed vascularization,^47^ however, it can be concluded that these vessels now allow cancer growth despite anti angiogenic treatment.

In a case report study of a renal cell carcinoma (RCC), Kats‐Urgurlu et al showed that lung metastases occurred 3 years after the primary tumor resection. Treatment with the antiangiogenic drug cedinarib and gefitinib, an epidermal growth factor receptor (EGFR) inhibitor was started and partial response was achieved. The metastatic lesions were resected but ultimately new lung metastases appeared. The authors reported that antiangiogenic treatment caused an adaptation of the phenotype in the lung with a “signature” infiltration of cancer cells along and even in the pulmonary vasculature, resembling pulmonary lymphangitis carcinomatosis, reflected by a cloudy pattern on CT, alongside nonangiogenic intra alveolar growth. These reports suggest that lung metastases of RCC can escape antiangiogenic treatments by switching phenotype and progress in an angiogenesis‐independent fashion exploiting preexisting vessels.^48^ A second case report form a patient with glioblastoma showed that, on biopsies taken after antiangiogenic treatment, a pattern of infiltration around the normal brain vessels is present.^45^

Nonangiogenic growth and spread in the lung has also been linked to resistance to surgical treatment.^49^ In nonangiogenic carcinomas growing in the lung, neoplastic cells can spread by moving from one alveolar cavity to the other trough the septa pores.^49^, ^52^ Yagi et al observed that patients in which this type of spreading is present, clusters of neoplastic cells are observed away from the main tumor and this may explain the higher rate of local recurrence in these patients.

## 6 ANIMAL MODELS: RESISTANCE TO THYROSINE KINASES INHIBITORS

A first group of models employs antiangiogenic drugs of the tyrosine kinases inhibitor (TKI) group.

Bridgeman et al investigated the effect of sunitinib on xenografts from three different cell lines from breast, colorectal renal cancer respectively. Sunitinib inhibited the growth of all the subcutaneous tumors and reduced the vascular density, whereas, the same treatment did not inhibit the growth of 4T1 breast cancer and C26 colorectal cancer lung metastases and had no effects on vessel density. A response, but only partial, was instead observed on RENCA generated lung metastases. Histological examination revealed that both the 4T1 and C26 lines are growing in the lung in a nonangiogenic fashion, contrary to their angiogenic growth in the subcutaneous tissue. Otherwise, the lung metastases of the RENCA renal carcinoma are prevalently angiogenic; however, the residual secondaries after sunitinib treatment, presented a switch toward the nonangiogenic growth pattern.^53^

Comparable results have been obtained in another model where response of the subcutaneous tumor, in this case orthotopic implantation in the mammary pad of the human breast carcinoma variant MDA‐MB‐231/LM2.4 line and their visceral metastases where challenged with sunitinib, Pazopanib or the anti‐VEGFA antibody DC101. All the three drugs where effective in treating established orthotopic primary tumors either as monotherapy or in association with Paclitaxel. They instead failed to show an effect, either as monotherapy or combination chemotherapy when used, after the resection of the mammary pad xenograft, to treat visceral metastases. No data are presented in this study as far as the vascularization of the resistant metastases is concern but the hypothesis that vascular co‐option could be involved it is discussed.^54^

A third study show that sunitinib accelerated metastatic tumor growth and decreased overall survival in mice receiving short‐term therapy in various metastasis assays. A faster metastatic process was identified in mice treated with sunitinib in advance of intravenous implantation of tumor cells, raising the possibility of a “metastatic conditioning” in multiple organs. As in the two studies discussed above, these observations of metastatic acceleration were in contrast to the demonstrable antitumor benefits obtained when the tumor cell lines were grown orthotopically as primary tumors and subjected to Sunitinib treatment. The authors, however, do not investigate where vascular co‐option is occurring,^55^ therefore, we can only say that nonangiogenic growth may be, but not necessarily, responsible for the resistance to treatment.

In an orthotopic hepatocellular carcinoma mouse model the implant is initially sensitive to sorafenib, another TKI blocking the VEGF pathway signaling, only to develop resistance after 1 month when an invasive phenotype develops and cancer cells switch to co‐option of sinusoidal and portal‐tract vessels in association with upregulation of epithelial mesenchymal transition (EMT)‐associated genes.^56^

Alongside their observations on patients with metastatic melanoma previously cited, Helfrich et al evaluated also the activity of another TKI, PTK/ZK (Vatalanib), in a MT/ret transgenic mice, which spontaneously develop melanoma with metastatic disease.^47^ Tumor development during PTK/ZK somministration in these mice was characterized by a mature intertumoral vascular network and stabilization of the vascular wall. The level of mural cell differentiation was considered as major mediator of blood vessels response to anti‐VEGF therapy. The authors do not discuss the possibility of nonangiogenic tumors co‐opting preexisting vessels but define these malignant lesions exploiting mature vessels as “low angiogenic active” tumors not responding to anti‐VEGF therapy.^47^

A model of melanoma metastatic to the brain was set up injecting Mel157 cell line in the carotid artery of Balb/c mice. This model was chosen as the authors had already described that it grows in a nonangiogenic way by co‐opting the brain vessels.^57^ Although angiogenesis can be effectively blocked by ZD6474, a VEGFR2 TKI with additional activity against EGFR, they observed sustained tumor progression via co‐option. The authors base their claims on the following observations: (a) large abnormal vessels observed in the mice receiving placebo are not present in the ZD6474 treated tumors; (b) in the placebo, but not in the ZD6474 treated tumors, endothelial upregulation of CD34 is observed; *glucose transporter* (GLUT), a characteristic marker of the blood‐brain barrier, is present in the vessels of the treated but not of the placebo mice; c) ZD6474 intratumor vessels are not leaky, contrary to the placebo‐ receiving ones; d) the endothelial cells in the tumors receiving antiangiogenic treatment are not proliferating, as assessed by stain for Ki67 proliferating marker.^58^

Treatment with cabozantinib, a c‐MET‐ and VEGFR2 tyrosine kinase inhibitor, of BALB/c nu/nu mice with orthotopic E98 glioblastoma xenografts lead to an improvement in overall survival. Cabozantinib blocked angiogenesis with a consequent increase of hypoxia in the angiogenesis‐dependent cancer regions with following vessel normalization.

However, tumors eventually became more infiltrative and escaped treatment via vessel co‐option.^59^

## 7 ANIMAL MODELS: RESISTANCE TO THERAPEUTIC ANTIBODIES

Using animal models Frentzas et al^41^ reported that it is vascular co‐option the cause for the resistance to antiangiogenic approaches as those described in the above clinical examples. As vascular co‐option is associated with increased cell motility, the authors dissected the role of a complex involved with the nucleation‐promoting factor (NPF) into the nucleation of actin filaments, leading to cell motility. The actin‐related protein 2 (ARP2) and the ARP3 complex are involved with the NPF into the nucleation of actin filaments, leading to increase in cell motility. It is well‐established that the ARP2/3 complex is one of the most important factors promoting invasion and metastases.^60^ Using immunohistochemistry, Frentzas et al observed that the expression of the ARPC3 component of the complex in the liver metastases is higher in the nonangiogenic tumors compared to the angiogenic, in a murine model of liver metastases using the cancer cell line HT29. After knocking down ARP2/3, impairing of the motility is achieved while the cells maintain their proliferating rate. Injected into the liver these cells still produced metastatic lesions but prevalently angiogenic with almost complete suppression of the nonangiogenic phenotype. The mice with metastases formed by HT29 wild type cells lesions, predominantly nonangiogenic, failed to responded well to the VEGFA inhibitory antibody B20‐4.1.1; this antibody was instead effective in reducing the neoplastic lesions in mice injected with the ARP2/3 negative‐HT29 cells. As the abrogation of motility suppresses co‐option and drives the cells to grow in an angiogenesis fashion, the resulting lesions are sensible to antiangiogenic compounds. It can be concluded that it is the nonangiogenic nature of the tumor causing resistance in this model and this can be reversed by stopping co‐option happening.^41^

A more recent study demonstrated that patient‐derived colorectal cancer organoids, grafted into the livers of mice, adopted a replacement growth pattern and that these tumors responded poorly to the antiangiogenic drug regorafenib^61^ confirming Frentzas findings.

Franco et al using a genetically engineered mouse model of pancreatic neuroendocrine tumors, found that tumors refractory to long‐term treatment with the anti VEGFR2 blocking antibody DC101, contained mature blood vessels covered by pericytes. Markers used to identify pericytes were alpha smooth actin (αSMA), NG2 proteoglycan and platelet‐derived growth factor receptor (PDGFR)‐beta. As in control animals, mature vessels covered by pericytes where at the edge between tumor and normal tissue and in the surrounding normal tissue, the authors concluded that the increased abundance of this particular subtype of blood vessels inside the resistant neoplasms, most likely occurred by co‐option of vessels from the surrounding exocrine pancreas.^62^

Murine and rat models are very well established models to study nonangiogenic growth and vascular co‐option.^63^, ^64^, ^65^, ^66^ VEGF secreting cell line G55, derived from human glioblastoma, was implanted in basal ganglia of nude rats creating an orthotopic model.^67^ Tumors examined before treatment exhibited high levels of neovascularization and grow more slowly as a consequence of antiangiogenic treatment, using a no better described anti‐VEGF antibody, leading to increased overall survival of the treated mice. However, post mortem examination revealed that while the primary tumors of treated animals had an increased level of apoptosis, they also had the histologic pattern of growth showing that they adapt to inhibition of angiogenesis by increased infiltration and co‐option of the host vasculature.^67^

That orthotopically implanted glioma cells invaded and proliferated within brain perivascular space was once more demonstrated using fractal dimension analysis on fluorescence scanning confocal micrographs of the orthotopic grafts.^68^ “In silico” simulation further confirmed that glioma cells can grow in absence of new vessels formation.

Perivascular invading brain tumors are irrorated by normal brain microvessels as individual glioma cells use perivascular space to invade. Both antibodies, bevacizumab and DC101, failed to reduce neoplastic growth or to improve overall survival of mice bearing wither orthotopic grafts or endogenous gliomas. Furthermore, the neoplastic cells become more invasive in treated animals. These results provide further experimental evidences that vascular co‐option by nonangiogenic tumors is a way to escape antiangiogenic treatment.^45^, ^68^

Others orthotopic murine model once again showed that in animals treated with either DC101, bevacizumab or VEGF‐Trap (a decoy molecule trapping VEGFA) confirmed that presence of nonangiogenic cancer cells can lead to tumor progression. Despite the observation of a marked reduction of the primary lesions, there was a striking increase in the number small perivascular lesions developing away from the primary mass. Such a satellite lesions were also recognizable distant from the primary due to an increased migratory ability of these nonangiogenic cells. Infiltration of the leptomeninges has been also observed. The authors concluded that blockage of the VEGFR2 can stop neovascularization and reduce the primary tumor but can, at the same time, lead to increased co‐option of preexistent cerebral blood vessels, disease spreading and therefore defeat the original object of achieve treatment.^44^, ^45^, ^69^, ^70^, ^71^, ^72^ Having reported that patients with lymph node metastatic cancer, treated with bevacizumab, have no benefits compared to the control group, Jeong et al set up an animal model in which they installed a chronic lymph node window which allowed to follow the development of the metastases and the effect of drugs in real time. Using multiphoton microscopy, they described how there are no evidences of sprouting of new vessels during the colonization of the lymph node and how antiangiogenic treatment with the DC101 antibody or with sunitinib fail to have any effect.^51^, ^73^

## 8 VASCULOGENIC MIMICRY

A few studies have started to explore the role of vasculogenic mimicry in inducing resistance to anti angiogenic treatment.

In an in vitro study van der Schaft et al^74^ found that three different antiangiogenic compounds, anginex, TNP‐470, and endostatin, were able to block “in vitro” formation of vascular channels using the human endothelial cells HUVEC and HMEC‐1. However, they could not prevent the human melanoma MUM‐2B and C8161 cells from forming tubular networks.

Xu et al^75^ injected the SKOV3 human ovarian cancer cell line into the tail vain of nude mice. Short‐term treatment with bevacizumab as single agent actually increased the number of lung metastases while both the two groups receiving cisplatin, with or without bevacizumab, had a similar level of regression. An increased number of vasculogenic mimicry channels has been found in tissue from the group treated with bevacizumab, alone or with cisplatin, although the authors do not specify the data according to the localization of metastases (lung, liver, or leg). These findings suggest that bevacizumab has, if any, a negative effect in this model, but negative consequences of bevacizumab are neutralized by cisplatin.

As vascular co‐option, also vascular mimicry plays a role in the resistance to anti angiogenic drugs in orthotopic glioblastoma models.^76^ Mice treated with the TKI Vatalanib^77^ had the volume of their tumors increase compared to the controls, as demonstrated with MRI and post mortem examination. Histopathological analysis also revealed an increase in vascular mimicry. No observations as far as the presence of vascular co‐option are provided. A third group of mice was treated with *N*‐hydroxy‐*N*′‐(4‐butyl‐2 methylphenyl) formamidine (HET0016), a highly selective inhibitor of 20‐hydroxy eicosatetraenoic acid (20‐HETE) synthesis, by regulating the enzymes of the cytochrome P450 families. This compound has been reported to be an effective angiogenesis inhibitor in preclinical models.^77^ Angora et al report that in this group there is also a reduction of vasculogenic mimicry and, in the group treated between 8 and 21 days, there is a significant shrinkage of lesions.^77^ In a subsequent study the same authors further illustrate that vasculogenic mimicry is one of the causes of resistance by illustrating that knocking down or the pharmacological inhibition of CXCR2, a mediator of vasculogenic mimicry, induces shrinkage of other ways resistant tumors.^78^

## 9 WHAT IS TO BE DONE?

**FIGURE 3:**
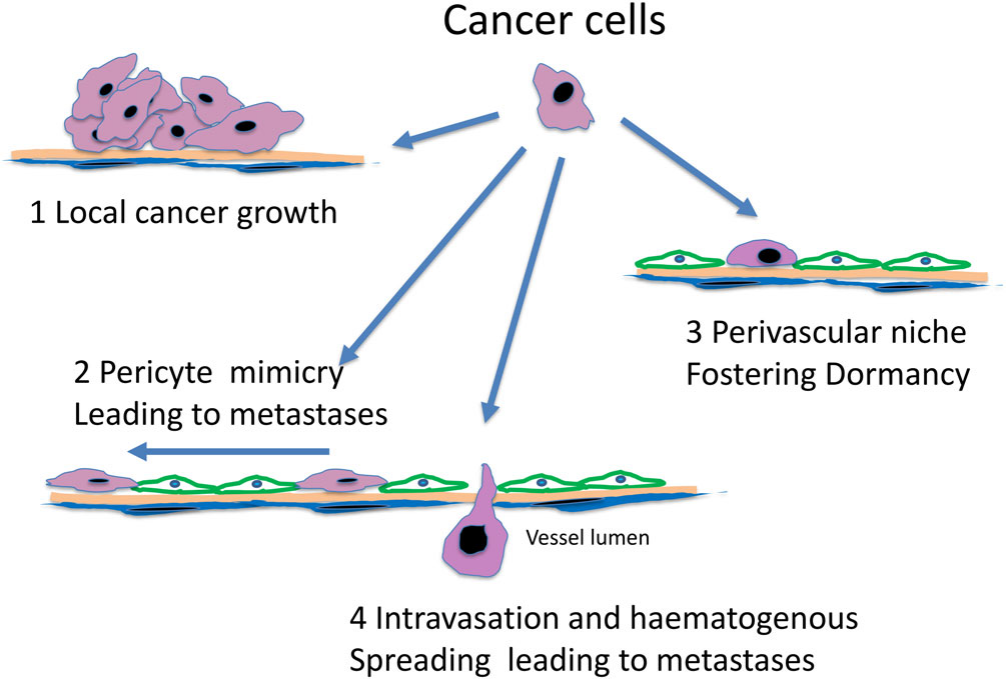
Post co‐option events and possible therapeutic strategies. Having co‐opted a vessel, the cancer cell can behave in different way. It can grow around the vessels (1) or instead, can acquire migratory property (pericyte mimicry) and, rather than grow, migrate along the abluminal surface (2) and eventually produce distant metastatic lesions. A third possibility is that a neoplastic cell, having co‐opted the vessels, will sit quiescent in a “perivascular niche” as a dormant cancer cell (3). As already well know some cells instead will immediately cross the blood vessels wall, enter the blood stream, and produce haematogenous metastases (4). Targeting the molecular apparatus that leads to co‐option is an obvious approach to block both local growth (1) and perivascular spread (2). Requiring even more work but holding more promises, is the possibility of preventing the formation of perivascular niches, disrupts the niche already formed or even just to prevent the existing dormant cells from “weak up” in patients with high risk of relapsing with metastatic disease. This approach is very likely to be more effective than trying to treat established metastatic lesions

So far antiangiogenic trials have been conducted mostly on the principle that all the tumors depend on neoangiogenesis and therefore any neoplastic patient has been automatically deemed eligible,^2^, ^12^ but need, is now emerging for predictive biomarkers and the new knowledge from nonangiogenic tumors and co‐option suggests new targets (Figure 3).

A first step in solving these issues would be to run clinical trials where patients are selected for antiangiogenic drugs according to predictive biomarkers.

An obvious one would be the vascular pattern of the tumors, however this at present require surgical resection to allow histological examination, but in most of the cases extensive surgery is not needed. Radiological imaging will hopefully provide with methodology alternative to the histopathological characterization. An example is the work of Cheng et al^79^ proposing that contrast‐enhanced multidetector CT scan imaging can be used to distinguish vessel co‐opting tumors from angiogenic tumors in patients with CRC liver metastases.

Another emerging approach is to combine antiangiogenic compounds with blockage of vascular co‐option. This is likely to be the most successful because tumors can actually change their vascular status during progression in both ways^9^, ^80^: an angiogenic tumor treated with antiangiogenic compounds can “escape” by turning nonangiogenic but a nonangiogenic tumor could “escape” anti‐co‐option drugs acquiring an angiogenic phenotype.

The combined approach is now being explored at preclinical level. The use of bevacizumab with the antibody OS2966 against beta1integrin, one of the keys molecules in co‐option, has demonstrated that the two have a synergic effect.^81^ Seaman et al^82^ observed that CD276, a highly conserved cell‐surface protein, is overexpressed in a variety of neoplastic cells and on intratumour endothelium both newly formed and trapped preexisting vessels. However, it is not present in normal vessels outside the neoplastic lesion or during physiological angiogenesis (eg vessels associated with inflammation). In their mouse model, a drug conjugated with anti CD276 antibody, was successful in targeting the tumors and improving long‐term overall.^82^

The potential value of this “double therapy” approach is further supported by a very thorough study by Voutouri et al^83^ combining intravital microscopy imaging and mathematical modeling to explore the dynamics of how individual neoplastic cells co‐opt vessels and how collective response of the co‐opted cancer cells during antiangiogenic treatment. The study included orthotopic models of glioblastoma and of breast cancer brain metastases treated with the Cedinarib, a pan‐VEGF RTKI. A mathematical model was developed which takes into consideration the mechanical pressure on co‐opted vessels, the subsequent hypoxia and the resulting formation of new vessels.

This model further indicates that vascular co‐option is a mechanism of escape and suggest that “double therapy,” antiangiogenic and anti‐co‐option, is predicted to give the best results.^83^

## 10 CE N'EST QU'UN DÉBUT (ON THE WALLS OF PARIS, MAY 1968)

As always, things in biology are frequently more complicated that initially tough. Deemed as one of the six original Hallmark of Cancer,^84^ the induction of angiogenesis it is actually not such an Hallmark. However, with all his limits and failures, the effort to treat tumors just by antiangiogenic therapy has nevertheless led to study with more attention the role of cancer in blood vessels. Once we will learn more about this relationship, both in angiogenic and nonangiogenic growth, hopefully we will be able to better exploit the antiangiogenic protocols so far designed alongside new treatments which are emerging by the study of vascular co‐option. Two are the main areas which promises to provide more targets for treatments. The first is the biology of co‐option as this is an active process requiring specific pathways while the second is the biology of the cancer cell when in nonangiogenic mode, as it differs in several aspects form the angiogenic ones.

Another potentially very important issue in cancer treatment, is the plasticity cancer cells can have: one example is how some neoplastic cell lines produce an angiogenic tumor if injected subcutaneous but a nonangiogenic one if eventually lodging into the lung after inoculation in the tail vein.^53^ This fact also suggest that there are not necessarily committed angiogenic or nonangiogenic tumors, but rather a tumor can have an angiogenic or nonangiogenic behavior as also suggested by clinical studies.^80^ Such a plasticity can therefore probably explain why some neoplastic cells, able to grow in some microenvironment, remain apparently “dormant” in a quiescent status after co‐opting a vessel.^85^ This perivascular microenvironment in which this happens has been called “the perivascular niche.” Should be confirmed that indeed these perivascular quiescent cells represent dormant disease, the knowledge of their biology could bring, if not to the ability to eliminate them, at least of preventing them from originating metastatic relapses.^86^ The recent advances in cancer biology due to the discovery of nonangiogenic growth have therefore the potential to lead to further steps toward a more effective cancer treatment.

## CONFLICT OF INTEREST

The authors declare that they have no conflicts of interest.

## ETHICAL STATEMENT

Not applicable.

## AUTHOR CONTRIBUTIONS

**Francesco Pezzella:** Conceptualization; writing‐original draft; writing‐review and editing. **Domenico Ribatti:** Conceptualization; writing‐original draft; writing‐review and editing.

## Data Availability

Data sharing is not applicable to this article as no new data were created or analyzed in this study.
